# An *ex vivo* Tissue Culture Model for the Assessment of Individualized Drug Responses in Prostate and Bladder Cancer

**DOI:** 10.3389/fonc.2018.00400

**Published:** 2018-10-02

**Authors:** Arjanneke F. van de Merbel, Geertje van der Horst, Maaike H. van der Mark, Janneke I. M. van Uhm, Erik J. van Gennep, Peter Kloen, Lijkele Beimers, Rob C. M. Pelger, Gabri van der Pluijm

**Affiliations:** ^1^Department of Urology, Leiden University Medical Center, Leiden, Netherlands; ^2^Department of Orthopedic Surgery, Amsterdam UMC, Amsterdam Movement Sciences, Amsterdam, Netherlands; ^3^Department of Orthopedic Surgery, MC Slotervaart, Amsterdam, Netherlands

**Keywords:** *ex vivo* tissue culture, prostate cancer, bladder cancer, compound testing, personalized medicine

## Abstract

Urological malignancies, including prostate and bladder carcinoma, represent a major clinical problem due to the frequent occurrence of therapy resistance and the formation of incurable distant metastases. As a result, there is an urgent need for versatile and predictive disease models for the assessment of the individualized drug response in urological malignancies. Compound testing on *ex vivo* cultured patient-derived tumor tissues could represent a promising approach. In this study, we have optimized an *ex vivo* culture system of explanted human prostate and bladder tumors derived from clinical specimens and human cancer cell lines xenografted in mice. The explanted and cultured tumor slices remained viable and tissue architecture could be maintained for up to 10 days of culture. Treatment of *ex vivo* cultured human prostate and bladder cancer tissues with docetaxel and gemcitabine, respectively, resulted in a dose-dependent anti-tumor response. The dose-dependent decrease in tumor cells upon administration of the chemotherapeutic agents was preceded by an induction of apoptosis. The implementation and optimization of the tissue slice technology may facilitate the assessment of anti-tumor efficacies of existing and candidate pharmacological agents in the complex multicellular neoplastic tissues from prostate and bladder cancer patients. Our model represents a versatile “near-patient” tool to determine tumor-targeted and/or stroma-mediated anti-neoplastic responses, thus contributing to the field of personalized therapeutics.

## Introduction

Urological cancers, including prostate and bladder cancer, represent a major global clinical problem. Prostate cancer is the most frequently diagnosed cancer type in men, whereas bladder cancer is the fifth most prevalent cancer type in the Western world (1, 2). The development of distant metastases and therapy resistance represent major clinical challenges in both carcinomas. Upon dissemination to distant organs, the 5-year survival of patients suffering from prostate and bladder cancer decreases dramatically (3). The current standard of care for advanced prostate and bladder cancer includes the use of chemotherapeutic agents, such as docetaxel and gemcitabine. (4–8). Previous studies have indicated that the response to chemotherapy in these patients is heterogeneous. A significant subset of the patients does not respond to chemotherapy or will develop resistance to this treatment (7, 9). Novel means of predicting individual therapy responses are, therefore, urgently required.

Another hurdle in the implementation of novel therapy for urological malignancies is the relatively low approval rate of candidate anti-tumor agents by the FDA and EMA (10). The latter can be attributed, in part, to the lack of predictive preclinical disease models. Current preclinical testing often neglects the importance of intra-tumor heterogeneity and the critical reciprocal interactions between the tumor cells and the cellular and acellular tumor-microenvironment (11).

By optimizing the collection and culture conditions, we have implemented an improved “near-patient” model that better allows compound testing on multicellular *ex vivo* cultured tumor tissues, either derived from explanted patient-derived primary and metastatic tumor tissues or patient-derived xenografts. The use of our tissue slice *ex-vivo* model will facilitate screening of the anti-tumor responses to established and candidate agents in individual patient-derived tumor tissues, either tumor-targeted or stroma-mediated anti-neoplastic agents. This will contribute to a more personalized therapeutic approach in patients with urological malignancies.

## Material and methods

### Animals

All animal experiments were performed after approval by the Animal Welfare Committee of the Leiden University Medical Centre (LUMC) (DEC14190 and DEC14212). Severe immunocompromised male NOD.Cg-Prkdc^Scid^Il2rg^tm1Wji^/Szj (NSG) mice were used for all xenografting experiments with human prostate cancer cells. Female nude mice (Balb-C nu/nu) were used for orthothopic inoculation of human bladder cancer cells. All mice were housed in individually ventilated cages under standard conditions in the animal facilities of the LUMC. Food and water were provided *ad libitum*.

### Xenografting of human prostate and bladder cancer cells

NSG mice were used for all xenografting experiments with the human osteotropic prostate cancer cell line PC-3M-Pro4. 250,000 PC-3M-Pro4 cells in 10 μl PBS were mixed with 10 μl growth factor-reduced Matrigel (BD Biosciences). Subsequently, the suspension was subcutaneously injected in the flank. In order to generate human bladder cancer xenografts, 5,000,000 UM-UC-3 cells were orthotopically inoculated in female immunodeficient mice (12).

Tumors were harvested and collected in medium supplemented with serum and antibiotics at room temperature. Explanted tumor tissues were processed for *ex vivo* culturing or directly fixed in 4% paraformaldehyde for 1 h at room temperature (described below).

### Cell culture

Human prostate cancer cells PC-3M-Pro4 were maintained in DMEM Glutamax, 4.5 g/L D-Glucose with Pyruvate (Gibco) supplemented with 10% FCII and 1% penicillin-streptomycin. Human bladder carcinoma cells UM-UC-3 were maintained in EMEM (ATCC) with 10% FCS and 1% penicillin-streptomycin. All cells were maintained in a humidified incubator at 37°C with 5% CO_2_.

### Viability assay

Treatment with a dose range ranging from 0.03 to 30 nM of docetaxel and 0.05–100 nM of gemcitabine was used to generate so-called “death curves” on a confluent layer of PC-3M-Pro4 and UM-UC-3 cells. After 72 h of treatment, 20 μl of 3-(4,5 dimethylthiazol-2-yl)-5-(3-carboxymethoxyphenyl)-2-(4-sulfophenyl)-2H-tetrazolium (MTT)(Promega) was added to each well (96-wells plate, Corning Costar). After 2 h, absorbance at 490 nm was measured (Spectramax plate reader).

### *Ex vivo* culture of tumor tissue slices

Tumor tissues were collected at room temperature and cut in ~1 mm^3^ pieces with forceps and scissors. Next, the tissue slices were placed on nitrocellulose filter inserts (6- well filter inserts, pore size of 4 μm, Corning Costar) positioned in 6-well culture plates. The culture plates were filled with 1 ml of culture medium (13). Prostate cancer tissues were cultured in DMEM Glutamax, 4.5 g/L D-Glucose with Pyruvate (Gibco) supplemented with 10% FCII or 10% FCS and 1% penicillin-streptomycin. Bladder cancer tissues were maintained in EMEM (ATCC) with 10% FCS and 1% penicillin-streptomycin.

**Figure 1:**
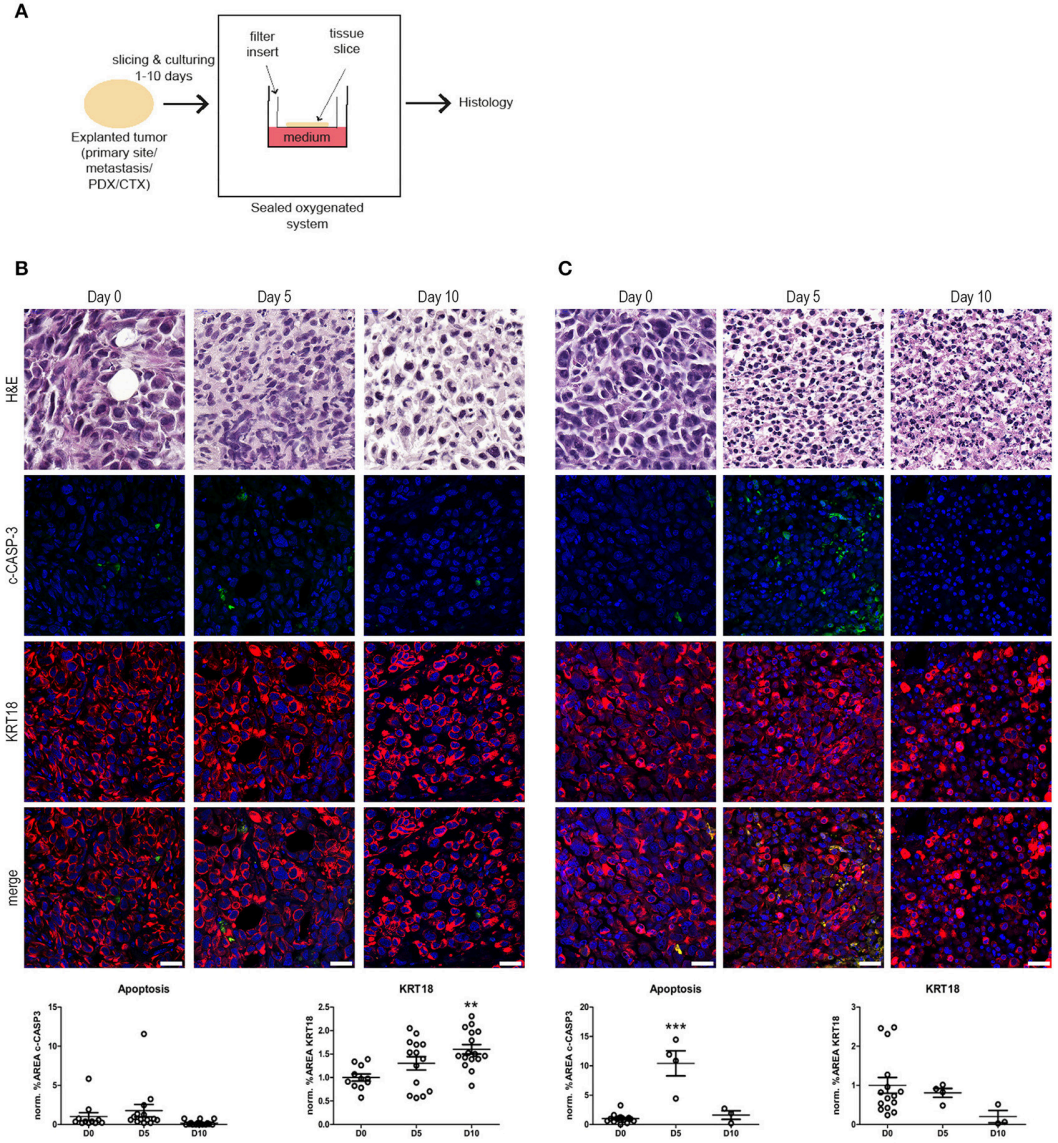
*Ex vivo* culture of prostate and bladder cancer tissue slices. **(A)** Schematic overview of the *ex vivo* culture system. Human tumor tissue (i.e., cell-line derived material or primary patient material) were manually processed in tissue slices. The tumor tissue slices were placed on a nitrocellulose filter insert that was placed in direct contact with cell culture medium in a culture plate. Subsequently, the tissue slices were cultured in a sealed and oxygenated system for 1–10 days and processed for histology. **(B)** Human prostate cancer tissue slices were generated from subcutaneous PC-3M-Pro4 xenografts in adult male NSG mice and subsequently cultured for 5 and 10 days (see Materials and Methods section). Culture of prostate cancer tissues resulted in the maintenance of tissue architecture for up to 10 days, indicated by intact nuclear morphology and normal KRT18 protein in intact, viable tumor cells. No significant changes in levels in cleaved caspase-3 (c-CASP-3) were detected upon culture. KRT18-positive tumor cells steadily increased during culture. Scale bar = 25 μm. **(C)** Human bladder cancer tissue slices were produced from orthotopically grown UM-UC-3 cells. Subsequently, the bladder cancer tissue slices were cultured for 5 and 10 days. Culturing of bladder cancer tissue *ex vivo* for 5 days resulted in a significant induction of apoptosis (*P* ≤ 0.001). KRT18-positive tumor cells steadily decreased during culture, but intact KRT18 protein could still be detected after 5 days of culturing. After 10 days of culture, nuclear fragmentation, and residual KRT18 protein were observed in apoptotic and dead tumor cells. Scale bar = 25 μm. **p < 0.01, ***p < 0.001.

The tissue slices were cultured in an oxygenated and sealed container system containing 95% O_2_ in the presence of docetaxel, gemcitabine, or vehicle solution. Previous *in vitro* viability assays have indicated the sensitivity of PC-3M-Pro4 and UM-UC3 cells for docetaxel (IC_50_ = 1.9 nM) and gemcitabine (IC_50_ = 17.7 nM), respectively (Supplementary Figures 1A,B). The tissue slices were treated with 0.3 and 3 nM docetaxel (Sigma-Aldrich), 10 and 100 nM gemcitabine (kindly provided by the Leiden University Medical Center's pharmacy) or vehicle solution (100% ethanol 3300 × diluted in medium for docetaxel, 5000 × diluted in medium for gemcitabine). After culturing the tissues, the tissue was harvested and processed for histological analyses (Figure 1A).

### Collection of surgical waste material

Primary and bone metastatic cancer tissue were obtained according to standard procedures. Regarding the primary prostate and bladder cancer tissue, material was obtained via transurethral resection. Clinical data can be found in Supplementary Table 1. Since this research was performed on “waste material,” consent for using the tissue for research purposes according to the Medical Research Involving Human Subjects Act (WMO) and approval by the local ethics committee is not required.

### Histology and immunofluorescence

Tumor tissue was fixed in 4% paraformaldehyde for 1 h at room temperature. After fixation, the tissue was dehydrated by incubation in a series of increasing concentrations of ethanol. Subsequently, the tissue was cleared in xylene and embedded in paraffin. Paraffin sections (5 μm) were made and mounted on SuperFrost Plus slides (Thermo Scientific) (13).

Hematoxylin and Eosin (H and E) staining was performed in order to assess general histology. For immunofluorescence stainings, the sections were deparaffinated by incubation in Histoclear (National Diagnostics) and antigen retrieval was performed by boiling in Antigen Unmasking Solution (Vector Labs) for 40 min. Sections were blocked in 1% Bovine Serum Albumin (BSA), dissolved in 0.1% PBS-Tween (PBST) for 30 min at room temperature and incubated with primary antibody, cleaved caspase-3 (rabbit polyclonal, 1:500 diluted, Cell Signaling Technologies #9661) and cytokeratin-18 (mouse monoclonal, 1:800 diluted Dako #M7010), and dissolved in PBST at 4°C overnight. Staining was visualized by Alexa fluor-conjugated secondary antibodies (1:250 Invitrogen, 90 min RT). Slides were mounted with ProLong-Gold antifade reagent containing DAPI according to manufacturer's protocol (Invitrogen).

### Microscopy and quantification

The H and E stained sections were scanned with the Pannoramic MIDI scanner and photomicrographs were taken by using the Caseviewer 2.0 software (3D Histech).

Fluorescent staining was visualized by confocal microscopy (Leica SP8 confocal microscope). For quantification, approximately 4 pictures per section were made (20x magnification, resolution 512 × 512 pixels). The mean area percentage fluorescence was quantified with ImageJ (National Institutes of Health) by using the threshold to define the positive area.

### Statistical analysis

Statistical analysis was performed with GraphPad Prism 6.0. In order to test for statistical difference, a one-way ANOVA with Bonferonni post-test was performed. Values are represented as mean ± SEM. *P*-values smaller than 0.05 were considered to be significant (^*^P < 0.05; ^**^P < 0.01; ^***^P < 0.001).

## Results

Xenografts from human prostate cancer cells (PC-3M-Pro4) and human bladder cancer cells (UM-UC-3) were generated in adult immunocompromised mice (12, 14, 15). Subsequently, the tissues were explanted, sliced, and cultured *ex vivo* for 5 or 10 days in order to investigate the effect of tissue culture on the tumor tissue integrity (Figure 1). *Ex vivo* culturing of the prostate cancer tissue resulted in maintenance of general tissue architecture and nuclear morphology for up to 10 days. No significant induction of apoptosis, as determined by immunofluorescent staining for cleaved caspase-3 (c-CASP-3), was observed after 5 and 10 days of culture compared to freshly isolated, non-cultured tissue. The surface area of cytokeratin-18 (KRT18)-positive tumor cells steadily increased upon culture, thereby representing an increase in viable tumor cells during *ex vivo* culture (Figure 1B).

Cultured bladder cancer tissue displayed maintenance of tissue integrity and architecture up to 5 days of culture, (Figure 1C). After 10 days of *ex vivo* culture, however, increased nuclear fragmentation and KRT18 protein degradation were observed indicative of gradual loss of tissue architecture beyond 5 days of culture. Immunolocalization of c-CASP-3 revealed a significant induction of apoptosis after 5 days of culture (*P* ≤ 0.001) resulting in a decline in tumor burden upon culturing. These observations suggest that human bladder cancer tissues can be cultured without detectable quality loss for at least 5 days. Culturing of the tissues *ex vivo* beyond 5 days resulted in a gradual overall deterioration of tissue architecture and morphology of the bladder cancer tissues.

**Figure 2:**
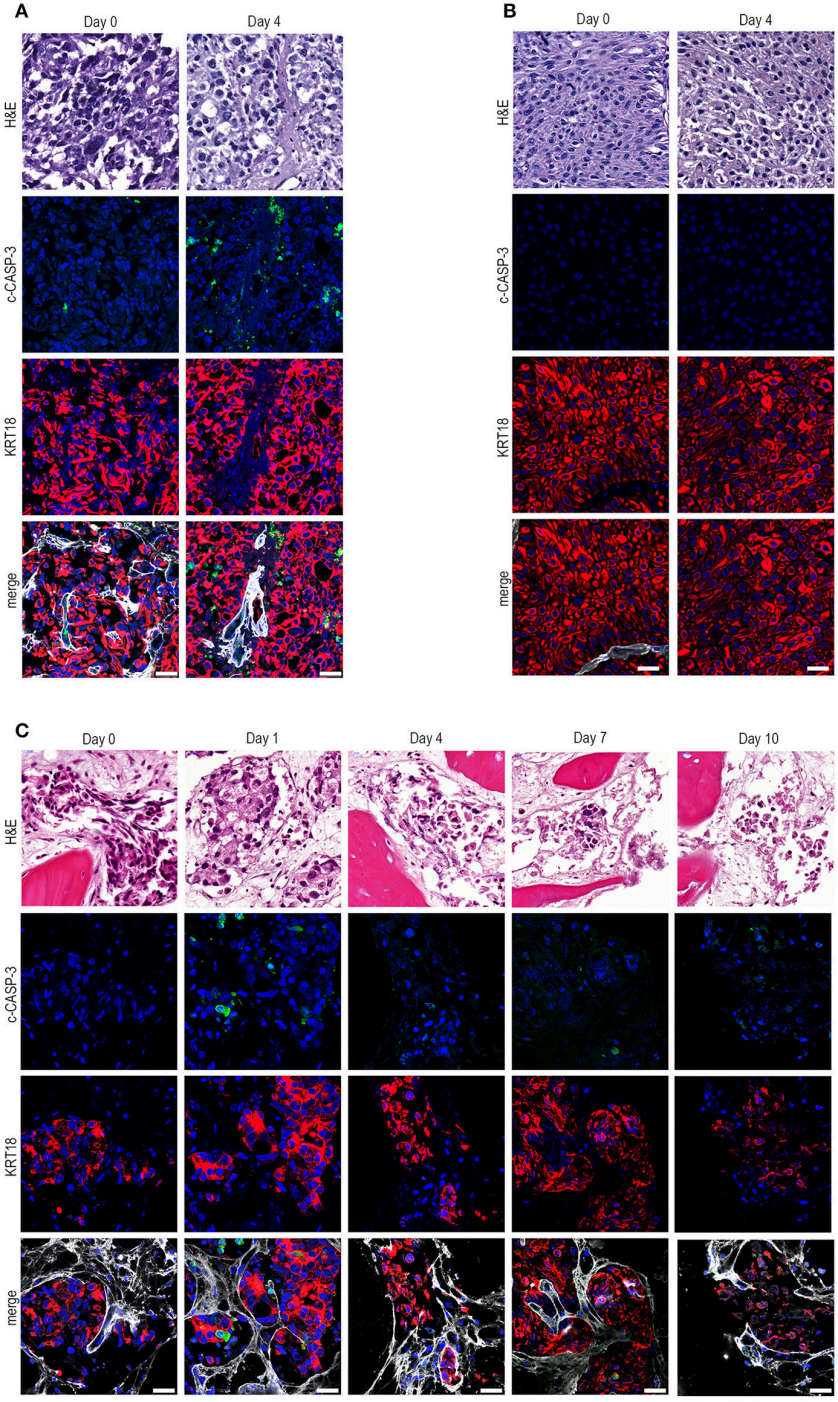
*Ex vivo* culture of prostate and bladder cancer patient-derived material. **(A)** Human prostate cancer tissue obtained after transurethral resection of the prostate (TURP) was cultured *ex vivo* for 4 days. *Ex vivo* culture resulted in intact tissue integrity and KRT18 staining after 4 days of culture. Scale bar = 25 μm. **(B)** Tumor tissue from a transurethral resection of the bladder (TURB) was obtained and cultured for 4 days. Tissue architecture was preserved, indicated by nuclear morphology, intact KRT18 staining, and absent c-CASP-3 staining. Scale bar = 25 μm. **(C)**. Tissue slices were generated from human prostate cancer bone metastasis material and cultured for 1, 4, 7, and 10 days. *Ex vivo* culturing for up to 4 days resulted in maintenance of tissue architecture, as indicated by intact nuclear morphology and normal KRT18 protein immunolocalization in neoplastic cells. Longer culturing (> 4 days) caused a gradual degradation of the tissue. Scale bar = 25 μm.

Next, we assessed whether our method could be used for *ex vivo* culturing of clinical prostate and bladder cancer specimens. Human prostate cancer tissue pieces were generated from material obtained after transurethral resection and cultured for 4 days (Figure 2A). *Ex vivo* culture resulted in intact tissue integrity and KRT18 staining after 4 days of culture, compared to uncultured tissue. Moreover, tumor material after transurethral resection of the bladder (TURB) from a non-muscle invasive bladder cancer patient was obtained and cultured for 4 days (Figure 2B). *Ex vivo* culture for 4 days resulted in the maintenance of normal tissue architecture, as indicated by normal nuclear morphology, intact membrane KRT18 staining, and absence of elevated levels of apoptosis (Figure 2B). Besides the culture of primary patient material, clinical metastasis material from an osteotropic prostate tumor was cultured for 1, 4, 7, and 10 days (Figure 2C). The prostate cancer pieces could be cultured for up to 4 days without significant loss of tissue integrity. Culturing beyond 4 days (day 7 and day 10) resulted in gradual deterioration of tissue architecture (Figure 2C).

Overall, these results indicate that our methodological adjustments allow compound testing in *ex vivo* cultures of explanted human prostate and bladder cancer tissue slices.

**Figure 3:**
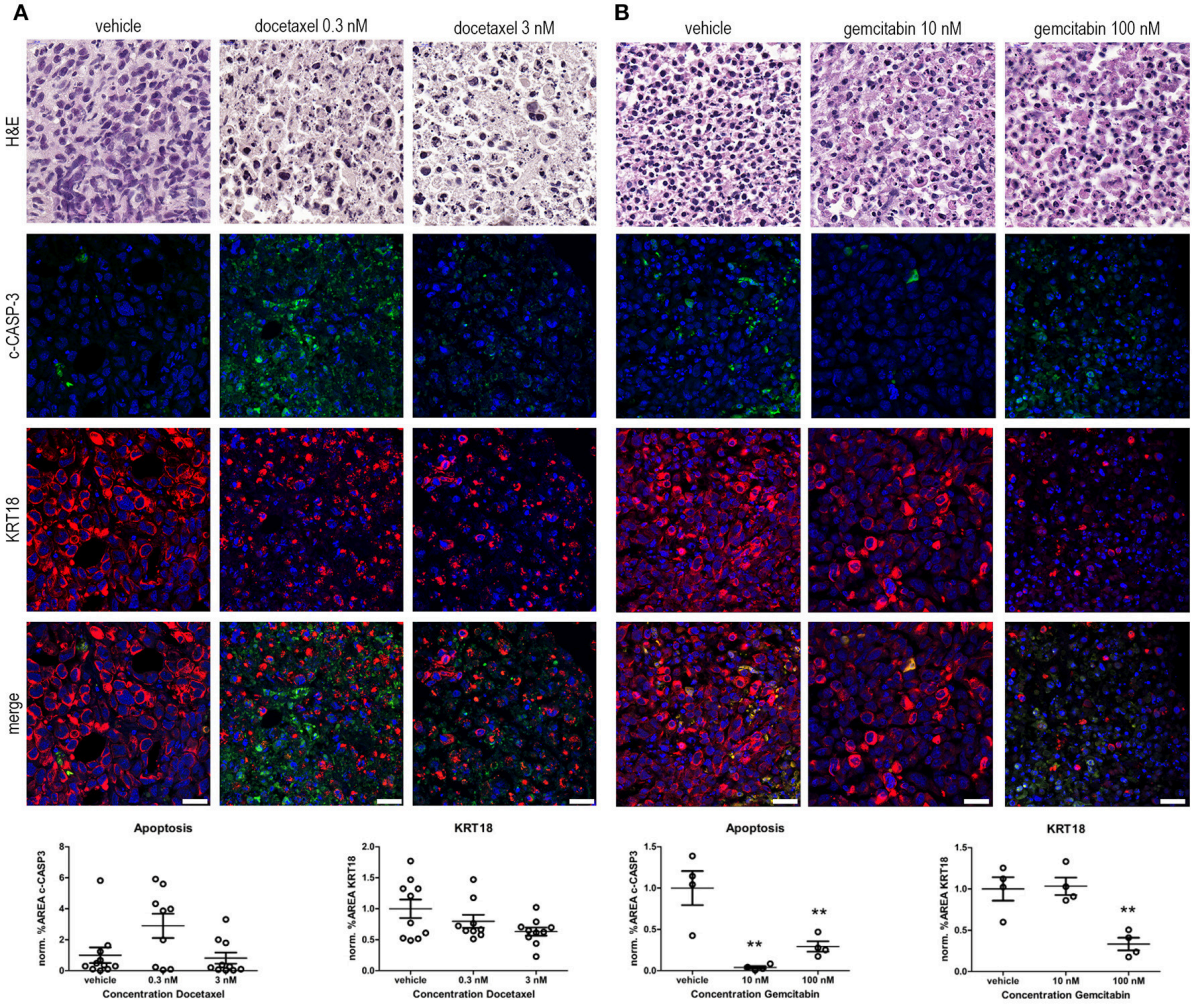
*Ex vivo* treatment of prostate and bladder cancer tissue slices with chemotherapeutic agents. **(A)** Prostate cancer tissue slices were *ex vivo* cultured in the presence of 0.3 and 3 nM docetaxel for 5 days. Treatment of the tissue slices with docetaxel resulted in an induction in c-CASP-3 levels and a decreased tumor burden, indicated by decreased KRT18 levels. Scale bar = 25 μm. **(B)** *Ex vivo* treatment of bladder cancer tissue slices with 10 and 100 nM gemcitabine for 5 days. Treatment of the tissue slices with 100 nM gemcitabine resulted in a significant decrease in KRT18 levels, accompanied with nuclear fragmentation. Strikingly, levels of c-CASP-3 were significant decreased upon gemcitabine treatment. Scalebar = 25 μm. **p < 0.01.

Subsequently, we examined whether the *ex vivo* culture technique could be exploited to assess therapy responses. For this, human prostate and bladder cancer tissue slices were generated and cultured in the presence of chemotherapeutic agents docetaxel or gemcitabine, respectively for 5 days (Figure 3A,3B).

Docetaxel (Taxotere) is mitotic spindle inhibitor and is frequently used as a treatment option in advanced prostate cancer. Treatment of the explanted, cultured human prostate cancer tissue with 0.3 nM docetaxel resulted in the induction of c-CASP-3 levels compared to vehicle treated tissue slices (Figure 3A). This was further substantiated by the presence of fragmented nuclei in the neoplastic cells in the docetaxel-treated tissue slices. In line with these observations a dose-dependent decrease in KRT18 immunolocalization was observed upon treatment with 0.3 and 3 nM docetaxel. Immunofluorescent and morphological analyses revealed degradation of KRT18 in the cancer cells indicative of decreased tumor cell viability and induction of cancer cell death.

Gemcitabine (Gemzar) is a nucleoside analog and is often used in combination with cisplatin as a treatment option for advanced bladder cancer. Treatment of the explanted, cultured bladder cancer tissues *ex vivo* with 100 nM gemcitabine (Figure 3B) significantly decreased the number of intact KRT18-positive tumor cells (*P* ≤ 0.01). Levels of c-CASP-3 were significantly reduced upon gemcitabine treatment (*P* ≤ 0.01 10 and 100 nM gemcitabine vs. vehicle). c-CASP-3 activity was significantly increased and was inversely related to KRT18 expression after 100 nM gemcitabine treatment compared to 10 nM. (both *P* ≤ 0.01). Overall, these results imply that our tumor tissue slice model can be exploited to monitor drug response in, explanted human prostate and bladder cancer tissues in an *ex vivo* setting.

## Discussion

Various preclinical models are currently applied in the oncological research field ranging from *in vitro* cell culture to patient-derived xenografting (PDX) (16). Obviously, each method has specific intrinsic limitations and cannot always be exploited for the assessment of potential therapy responses in individual patients. Currently available *ex vivo* tissue culture methods may suffer from a number of technical drawbacks and limitations. One impediment of existing *ex vivo* tissue culture systems is that viability and integrity of the tissue can generally not be maintained long enough to assess personalized therapy responses to existing or novel pharmacologic treatments. This may be due to, at least in part, tissue handling, and explantation methods (e.g., collection of the samples, temperature, and collection medium) and *ex vivo* culture conditions (e.g., submerged or on filter support, oxygen tension, poor diffusion of oxygen, and nutrients). Different tissue slice culture methods have been described in the literature, including submerging the tissue in culture medium, dynamic culturing with the use of rotating platforms and the use of mechanical support (16, 17).

In this study, we have developed an improved *ex vivo* culture method for explanted urological tumors (human prostate and bladder cancer tissues). Optimization of the culture conditions by collection of the tissue at room temperature, culturing on filter inserts under hyperoxic conditions has led to better preservation of the tissue architecture. The *ex vivo* method described in this study uses filter insert in order to direct mechanical support of the tumor tissue with the culture medium. Moreover, culturing of the tumor tissue under hyperoxic conditions resulted in an improved tissue integrity. These beneficial effects of culturing under hyperoxia could be explained by the fact that the increase in oxygen supply partly compensates for the lack of a functional blood supply and lymphatic vasculature in the cultured tumor tissues. It is important to note that the supply of oxygen and nutrients during tissue slice culture takes place by diffusion alone and is not further maintained by an active blood circulatory system.

Despite this, the tissue architecture and the heterotypic interactions between tumor cells and the supportive (a)cellular stroma are largely maintained for an extended period under hyperoxic conditions, thus creating a larger window of opportunity for compound testing. This was demonstrated in this study for the chemotherapeutic agents docetaxel and gemcitabine for human prostate and bladder tissues respectively.

Hence, the *ex vivo* tissue culture technology may provide a versatile platform for testing compounds directly on patient-derived tumor tissue or xenografted tissue. In this way, *ex vivo* tissue culturing may facilitate a more personalized therapeutic approach for individual cancer patients. Furthermore, the use of patient-derived tissues for assessing individual responses to pharmacological agents may provide a useful alternative to the often challenging xenografting of human tissue in immunocompromised mice (18–21). Moreover, other parameters within a single tissue slice can be examined upon *ex vivo* treatment, including gene and protein expression levels, proliferative and apoptotic responses. Although not described here, our “near-patient” model allows for the determination of putative therapeutic effects of compounds on the tumor compartment, the supportive stromal microenvironment or both. It should be noted that cancer tissue is not homogeneous and intra-tumoral heterogeneity is a complicating factor in studies aiming at addressing pathogenic mechanisms of tumor progression, therapy response, drug resistance, and for novel drug development (22–24). Moreover, the availability of tumor tissue remains an issue to fully examine the tumor material. The use of biopsy material in our “near-patient” model may not always represent the full heterogeneity within the primary tumor or the selected metastases. It may, therefore, be that the yet “unidentified lethal clone” of cancer cells is not always represented. It has been described previously in human prostate cancer that an area of Gleason pattern 3, thus indicating that a small, low-grade focus of prostate cancer may harbor an unrecognized killer (25, 26). Moreover, rare subclones of prostate cancer cells acquire metastatic properties within the primary tumor rather than the notion that metastatic potential is a property of the primary tumor as a whole (27).

An advantage of the *ex vivo* tumor tissue model is that the differential efficacy of anti-cancer treatments can be monitored in the various cancer subpopulations e.g., stem-like vs. more differentiated cancer cells. This may facilitate the discovery of candidate drugs that strongly target highly malignant tumor subclones.

Taken together, we have described a new method for *ex vivo* culturing of primary patient material or xenograft-derived human tumor tissue. This technique can be exploited for the assessment of personalized therapy responses to clinically-approved or candidate pharmacological compounds. It seems that our *ex vivo* tissue culture model opens a new window of opportunity and may represent a translational tool for determining the sensitivity of explanted, patient-derived tumor tissue for FDA- or EMA-approved therapeutic agents or for evaluating novel candidate drugs or the feasibility of drug repositioning.

## Author contributions

AvdM designed and performed experiments, acquired and analyzed the data, and wrote the manuscript. GvdH designed and performed experiments, acquired, and analyzed data. MvdM performed experiments and acquired data. JvU, EvG, PK, LB, and RP provided patient material. GvdP coordinated the study and wrote the manuscript. All authors contributed to reviewing and revising the manuscript.

### Conflict of interest statement

The authors declare that the research was conducted in the absence of any commercial or financial relationships that could be construed as a potential conflict of interest.
