# Supplementary material for: An ex vivo Tissue Culture Model for the Assessment of Individualized Drug Responses in Prostate and Bladder Cancer
Source: Front Oncol. 2018 Oct 2;8:400. doi: 10.3389/fonc.2018.00400 (PMC6176278; doi:10.3389/fonc.2018.00400)
Supplement: Supplementary file 1 [file Table_1.DOCX]

| **sample** | | **type** | **pathology diagnosis** | **tumor site** | **date of diagnosis** | **TNM stage at diagnosis** | **grade at diagnosis** | **PSA at diagnosis** | **treatment history before sample** | **date of sample** | **TNM stage at sampling date** | **grade at sampling date** | **PSA at sampling date** |
| --- | --- | --- | --- | --- | --- | --- | --- | --- | --- | --- | --- | --- | --- |
| 1 | prostate | | adenocarcinoma | prostate | Nov 2011 | T3N0M0 | Gl 3+4 | 6.9 µg/L | EBRT, LHRH agonist, Enzalutamide | Sep 2016 | CRPC: T4N+M+ | Gl 4+4 | 52.5 µg/L |
| 2 | prostate | | adenocarcinoma | prostate | May 2017 | T3bNxMx | Gl3+4 | 7.5 µg/L | LHRH agonist | June 2017 | T3bNxMx | Gl 3+4 | 7.5 µg/L |
| 3 | prostate | | adenocarcinoma | prostate | April 2017 | T2bNxMx | Gl4+4 | 62.9 µg/L | Androgen deprivation | July 2017 | T3bN0Mx | Gl 4+4 | 15.0 µg/L |
| 4 | prostate | | adenocarcinoma | prostate | April 2014 | T3bN+M0 | Gl 4+4 | 67.0 µg/L | LHRH agonist, Enzalutamide | Nov 2017 | CRPC: T3bN+M+ | Gl 5+5 | 1.58 µg/L |
| 5 | prostate | | adenocarcinoma | bone | March 2013 | T4N1M1 | Gl 9 | 54.0 µg/L | LHRH agonist, EBRT, Docetaxel | June 2016 | n.a. | n.a. | 580.40 µg/L |
| 6 | bladder | | urothelial cell carcinoma | bladder | Oct 2017 | Ta N0M0 | low grade | n.a. | none | Oct 2017 | Ta N0M0 | low grade | n.a. |
| 7 | bladder | | urothelial cell carcinoma | bladder | June 2015 | T1 N0 M0 | high grade | n.a. | none | June 2015 | T1 N0 M0 | high grade | n.a. |
| 8 | bladder | | urothelial cell carcinoma | bladder | Oct 2017 | pT1 | high grade and CIS | n.a. | BCG bladder instillations | Oct 2017 | pT1 | high grade and CIS | n.a. |
| 9 | bladder | | urothelial cell carcinoma | bladder | Nov 2015 | pT2 NxM0 | infiltrating , nested variant | n.a. | EBRT | Dec 2017 | pT2 N0 M0 | high grade | n.a. |
| 10 | bladder | | urothelial cell carcinoma | bladder | Dec 2017 | pTa N0 M0 | low grade | n.a. | none | Dec 2017 | pTa N0 M0 | low grade | n.a. |

Supplementary table 1 **Clinical histopathological characteristics**.

TNM stage, grade and PSA levels are shown at time of diagnosis as well as sampling date of the tumor material. Moreover, known treatment history of the patients is shown. Abbreviations: Gl: Gleason grade; CIS carcinoma in situ; PSA prostate specific antigen; EBRT external beam radiation; LHRH luteinizing hormone-releasing hormone; BCG Bacillus Calmette-Guerin; CRPC castration resistant prostate cancer; n.a. not applicable.
