# Supplementary figures and images for: An ex vivo Tissue Culture Model for the Assessment of Individualized Drug Responses in Prostate and Bladder Cancer
Source: Front Oncol. 2018 Oct 2;8:400. doi: 10.3389/fonc.2018.00400 (PMC6176278; doi:10.3389/fonc.2018.00400)

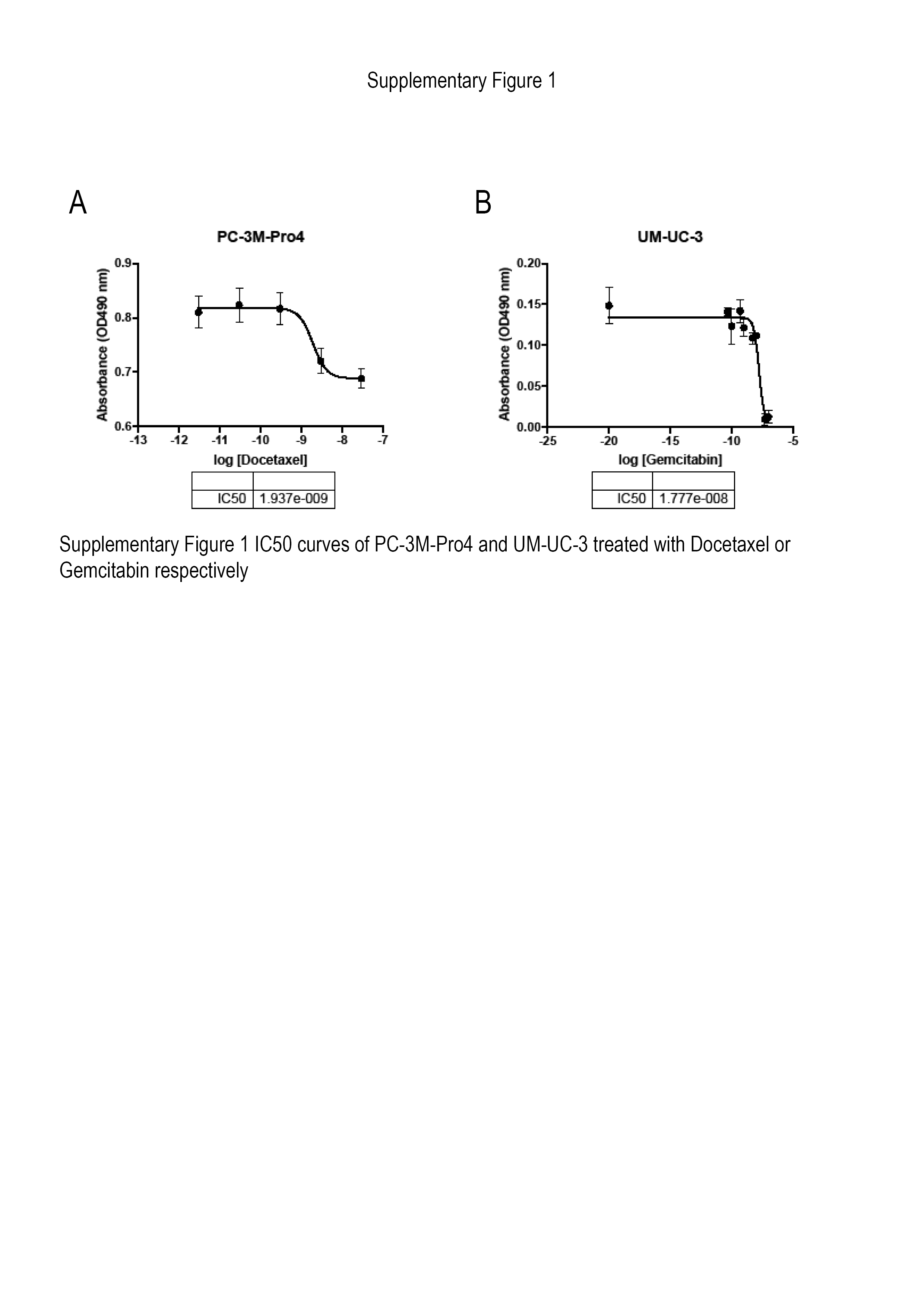

Supplement: Supplementary file 2 [file Image_1.jpg]
